# Supplementary figures and images for: Indoor climbing and well-being of young adults: Perspectives among indoor climbers
Source: PLoS One. 2025 Apr 29;20(4):e0321542. doi: 10.1371/journal.pone.0321542 (PMC12040214; doi:10.1371/journal.pone.0321542)

**Appendix 1:** *Interview Script*


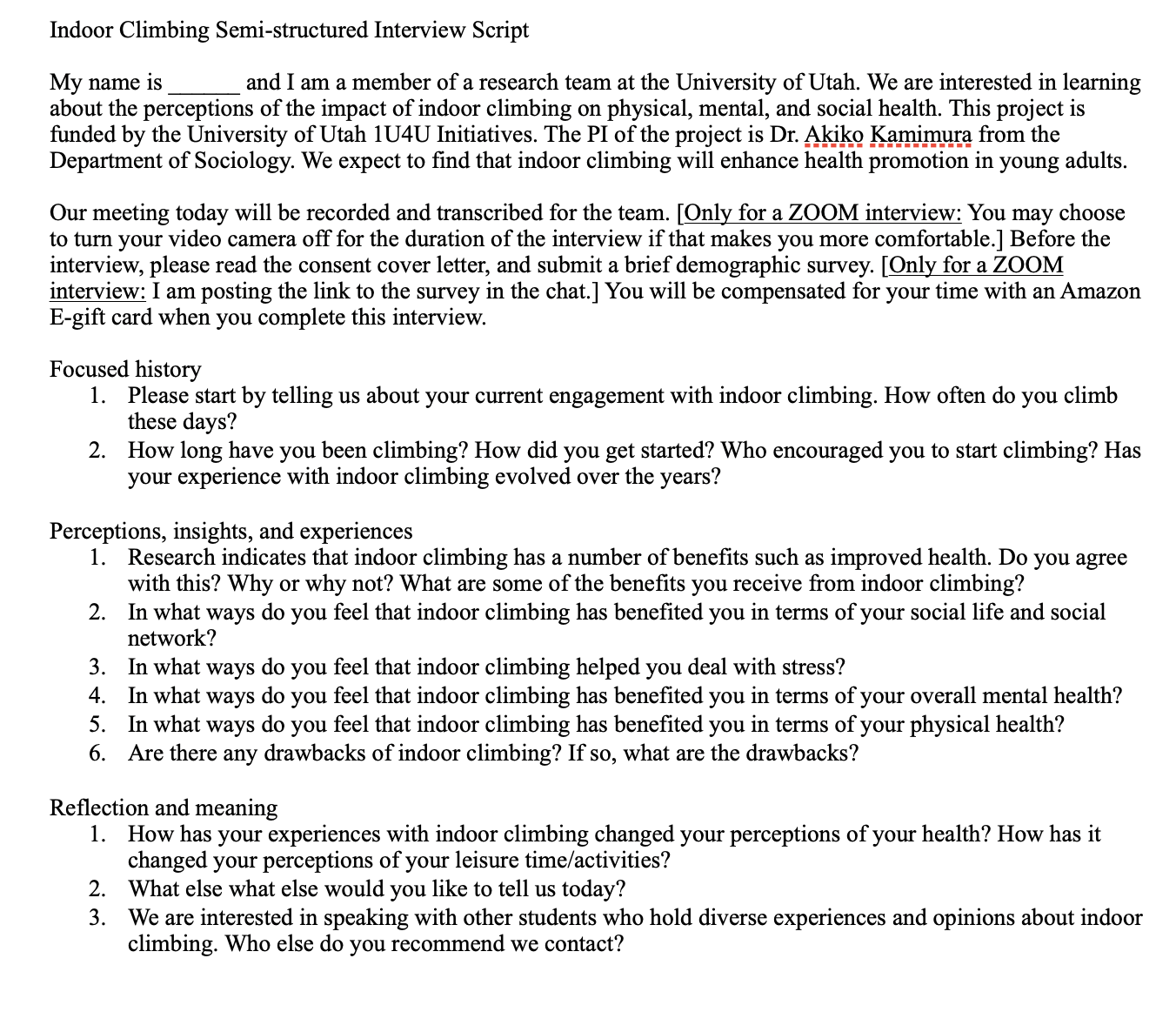

Supplement: S1 Appendix — (DOCX) [file pone.0321542.s001.docx]
