# Supplementary material for: Indoor climbing and well-being of young adults: Perspectives among indoor climbers
Source: PLoS One. 2025 Apr 29;20(4):e0321542. doi: 10.1371/journal.pone.0321542 (PMC12040214; doi:10.1371/journal.pone.0321542)
Supplement: Attachment — Submitted filename: comment article 2.docx [file pone.0321542.s002.docx]

PONE-D-24-41965

Indoor Climbing and Well-being of Young Adults: Perspectives among indoor climbers

**Abstract**: To summarize, refer to the statistical method

**Introduction:** Strengthen theoretical foundations (both positive and negative research)

**Methods:** It would have been better if people were selected from different regions

Data Analysis: In this section, refer to the data analysis in thematic form

**Discussion:** In the section rejecting or confirming proposals, strengthen the theoretical foundations with the results of your work
