# Supplementary material for: Indoor climbing and well-being of young adults: Perspectives among indoor climbers
Source: PLoS One. 2025 Apr 29;20(4):e0321542. doi: 10.1371/journal.pone.0321542 (PMC12040214; doi:10.1371/journal.pone.0321542)
Supplement: Attachment — Submitted filename: reviwe fail-24-41965.docx [file pone.0321542.s003.docx]

**Review of the Article: "** **Indoor Climbing and Well-being of Young Adults:
Perspectives among indoor climbers"**

Dear Authors,

To enhance the quality of this article, I kindly suggest the following revisions:

**Abstract:**

Please ensure the following points are addressed in the abstract:

- The introduction is too lengthy; it would be better if it is shortened.
- The objective related to the title should be clearly stated.
- The type of research and the research method should be mentioned.
- The coding approach and the method of analyzing the data from the interviews should be outlined.
- The conclusion should correspond with the findings.

**Introduction**:

- The introduction requires rewriting.
- Please revise the introduction to transition from a thesis style to an article format.
- Avoid including unnecessary headings in the introduction.
- Ensure coherence and consistency throughout the content.
- Present the introduction as a single section, starting with the main variables of the research, discussing the challenges and the need for the study, referencing related studies and their limitations, and finally stating the main objective or hypothesis.

**Research Methodology:**

- Provide a thorough explanation of the research method and type at the beginning of this section. Follow this with details regarding the participants and the sampling method used, and conclude with the execution and data analysis process. Write this section in line with article standards, avoiding a thesis-like style.
- Clarify whether coding was conducted during the interviews and explain how saturation was achieved.
- Considering that your sample comprised students, justify the use of a snowball sampling method.
- Include a detailed description of the interview questions in the methodology section.
- Explain how credibility, transferability, and dependability—key aspects of qualitative studies—were addressed.
- Ensure inter-rater reliability (coder reliability) is established, and report the results.

**Results**:

- Given the length of this section, avoid unnecessary details and focus on the main and subcategories.
- Present the main and subcategories, along with the extracted concepts, in tables.
- If coding was performed during the interviews, include a table reporting the interview codes.
- To aid understanding, provide a conceptual model illustrating the research findings.

**Discussion**:

- Revise the discussion to align with the research findings.
- Start by presenting the research findings, compare them with previous studies, and conclude with a comprehensive summary.
- Mention the limitations of the research and offer practical suggestions.

**Final Remarks:**

Your research topic is very interesting. However, more effort is needed to strengthen the writing in all sections to improve the overall quality and presentation of the article.
