# Supplementary material for: Indoor climbing and well-being of young adults: Perspectives among indoor climbers
Source: PLoS One. 2025 Apr 29;20(4):e0321542. doi: 10.1371/journal.pone.0321542 (PMC12040214; doi:10.1371/journal.pone.0321542)
Supplement: Attachment — Submitted filename: Response to the reviewers RR1 020425.docx [file pone.0321542.s004.docx]

Response to the reviewers

Reviewer 1

As you know, different parts of the article need corrections, and in order to edit and complete them, you need to read and correct the mentioned items in different parts. Different sections include abstract. Introduction. method Results. Discussion

Response: Thank you for your comments. We made revisions below.

Reviewer – one of the attachments

Abstract: To summarize, refer to the statistical method

Response: This is a qualitative study and data analysis was not based on statistical analysis. We clarified the methodological approach.

Introduction: Strengthen theoretical foundations (both positive and negative research)

Response: We revised the paragraph about the theory to make the theoretical foundation clearer.

Methods: It would have been better if people were selected from different regions

Response: Great point. We added it to the limitation section.

Data Analysis: In this section, refer to the data analysis in thematic form

Response: We presented it in Table 2 and made it clearer in the method section.

Discussion: In the section rejecting or confirming proposals, strengthen the theoretical foundations with the results of your work

Response: We included that point in conclusion.

________________________________________

Reviewer 2

Review of the Article: " Indoor Climbing and Well-being of Young Adults: Perspectives among indoor climbers"

Dear Authors,

To enhance the quality of this article, I kindly suggest the following revisions:

Abstract:

Please ensure the following points are addressed in the abstract:

The introduction is too lengthy; it would be better if it is shorten.

Response: We shortened it.

The objective related to the title should be clearly stated.

Response: Done

The type of research and the research method should be mentioned.

Response: Done

The coding approach and the method of analyzing the data from the interviews should be outlined.

Response: Done

The conclusion should correspond with the findings. Response: We revised the conclusion.

Introduction:

The introduction requires rewriting.

Response: We revised the introduction.

Please revise the introduction to transition from a thesis style to an article format.

Response: We revised the introduction.

Avoid including unnecessary headings in the introduction.

Response: We dropped the sub-headings.

Ensure coherence and consistency throughout the content.

Present the introduction as a single section, starting with the main variables of the research, discussing the challenges and the need for the study, referencing related studies and their limitations, and finally stating the main objective or hypothesis.

Response: We made the introduction to be a single section and followed the structure that you suggested. However, because this is a qualitative study, there is no hypothesis to present.

Research Methodology:

Provide a thorough explanation of the research method and type at the beginning of this section. Follow this with details regarding the participants and the sampling method used, and conclude with the execution and data analysis process. Write this section in line with article standards, avoiding a thesis-like style. Response: Thank you for your comment

Response: Thank you for your comments. We revised the method section.

Clarify whether coding was conducted during the interviews and explain how saturation was achieved. Response: Coding was conducted AFTER the interviews.

Considering that your sample comprised students, justify the use of a snowball sampling method.

Response: We justified the snowball sampling method.

Include a detailed description of the interview questions in the methodology section.

Response: We included more details about the in the description of the interview questions.

Explain how credibility, transferability, and dependability—key aspects of qualitative studies—were addressed.

Response: We added the information.

Ensure inter-rater reliability (coder reliability) is established, and report the results.

Response: We added the information.

Results:

Given the length of this section, avoid unnecessary details and focus on the main and subcategories.

Response:

Present the main and subcategories, along with the extracted concepts, in tables.

Response: It’s presented in Table 2.

If coding was performed during the interviews, include a table reporting the interview codes.

Response: Coding was not performed during the interviews.

To aid understanding, provide a conceptual model illustrating the research findings.

Response: We added Figure 1 that illustrates a conceptual model of the findings.

Discussion:

Revise the discussion to align with the research findings.

Start by presenting the research findings, compare them with previous studies, and conclude with a comprehensive summary.

Response: We re-organized the discussion section.

Mention the limitations of the research and offer practical suggestions.

Response: We added practical suggestions to the limitation section.

Final Remarks:

Your research topic is very interesting. However, more effort is needed to strengthen the writing in all sections to improve the overall quality and presentation of the article.

Response: Thank you for your comments. We hope that the paper has improved.
